# Understanding the complex mechanisms of β_2_-microglobulin amyloid assembly

**DOI:** 10.1111/j.1742-4658.2011.08186.x

**Published:** 2011-10

**Authors:** Timo Eichner, Sheena E Radford

**Affiliations:** 1 Department of Biochemistry, Brandeis UniversityWaltham, MA, USA; 2 Astbury Centre for Structural Molecular Biology and Institute of Molecular Cellular Biology, University of LeedsUK

**Keywords:** amyloid, conformational conversion, dialysis-related amyloidosis, dynamics, NMR, prion

## Abstract

Several protein misfolding diseases are associated with the conversion of native proteins into ordered protein aggregates known as amyloid. Studies of amyloid assemblies have indicated that non-native proteins are responsible for initiating aggregation *in vitro* and *in vivo*. Despite the importance of these species for understanding amyloid disease, the structural and dynamic features of amyloidogenic intermediates and the molecular details of how they aggregate remain elusive. This review focuses on recent advances in developing a molecular description of the folding and aggregation mechanisms of the human amyloidogenic protein β_2_-microglobulin under physiologically relevant conditions. In particular, the structural and dynamic properties of the non-native folding intermediate I_T_ and its role in the initiation of fibrillation and the development of dialysis-related amyloidosis are discussed.

## The role of β_2_-microglobulin in amyloid disease

**Fig. 1:**
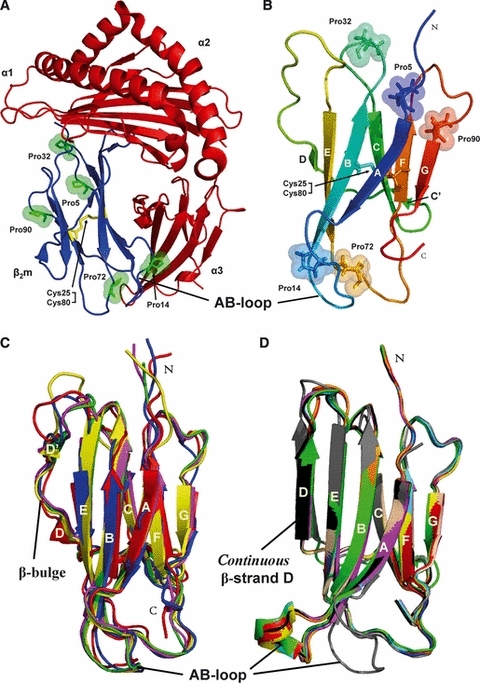
Monomeric β_2_m plays a key role in DRA. (A) Cartoon representation of human MHC I (PDB code 3MYJ [136]) showing the heavy chain (α1, α2, α3 in red) and the light chain (β_2_m in blue). Highlighted are the residues Pro5, Pro14, Pro32, Pro72 and Pro90 (in green sticks, spheres) and the disulfide bond between residues Cys25 and Cys80 (in yellow sticks). (B) Cartoon representation of the solution structure of monomeric native wild-type β_2_m (PDB code 2XKS [9]) showing β-strands A (6–11), B (21–28), C (36–41), C′ (44–45), D (50–51), E (64–70), F (79–83) and G (91–94). Highlighted are the residues Pro5, Pro14, Pro32, Pro72 and Pro90 (in sticks, spheres) and the disulfide bond between residues Cys25 and Cys80 (in sticks). N, N-terminus; C, C-terminus. (C) Structures displaying a β-bulge and an attached AB-loop: wild-type β_2_m (PDB code 1JNJ [7]) in red, H31Y (PDB code 1PY4 [15]) in green, W60G (PDB code 2VB5 [16]) in blue, H13F (PDB code 3CIQ [55]) in yellow and MHC I (PDB code 3MYJ [136]) in magenta. (D) Structures displaying a straight β-strand D: wild-type β_2_m (PDB code 1LDS [11]) in red, L39W/W60F/W95F (PDB code 2D4D [137]) in green, wild-type β_2_m (PDB code 2D4F [137]) in blue, wild-type β_2_m (PDB code 2YXF [12]) in yellow, W60G (PDB code 2Z9T [16]) in magenta, W60C (PDB code 3DHJ [14]) in cyan, D59P (PDB code 3DHM [14]) in orange, W60G (PDB code 3EKC [14]) in wheat, K58P/W60G (PDB code 3IB4 [121]) in black and P32A (PDB code 2F8O [58]) in grey.

β_2_-microglobulin (β_2_m) is the non-covalently bound light chain of the major histocompatibility complex class I (MHC I), wherein the protein plays an essential role in chaperoning assembly of the complex for antigen presentation (Fig. 1A) [1–3]. Wild-type β_2_m contains 99 amino acids and has a classical β-sandwich fold comprising seven anti-parallel β-strands that is stabilized by its single inter-strand disulfide bridge between β-strands B and F (Fig. 1B) [4–6]. The high resolution structures of monomeric native β_2_m from humans and several of its variants have been solved by solution NMR [7–10] and X-ray crystallography [4,11–16]. β_2_m contains five peptidyl–prolyl bonds, one of which (His31-Pro32) adopts the thermodynamically unfavoured *cis*-isomer in the native state (Fig. 1B) [4,7,9]. Another interesting feature of monomeric native β_2_m is the conformational dynamics of the D-strand and the loop that connects the D- and E-strands (the DE-loop) (Fig. 1B). This region forms contacts with the MHC I heavy chain [17], but shows dynamics on a microsecond to millisecond timescale when a monomer in solution [7] and variability in different crystal structures (Fig. 1C,D) [13]. This rationalizes hydrogen–deuterium exchange studies on monomeric native β_2_m showing that the DE-loop region exhibits enhanced backbone dynamics compared with the non-covalently MHC I bound state [18]. Notably, a link between the dynamic properties of monomeric native β_2_m, particularly in the D-strand and the DE-loop region, and its potential to assemble into amyloid fibrils has been proposed [7,10,11,18–20].

**Fig. 2:**
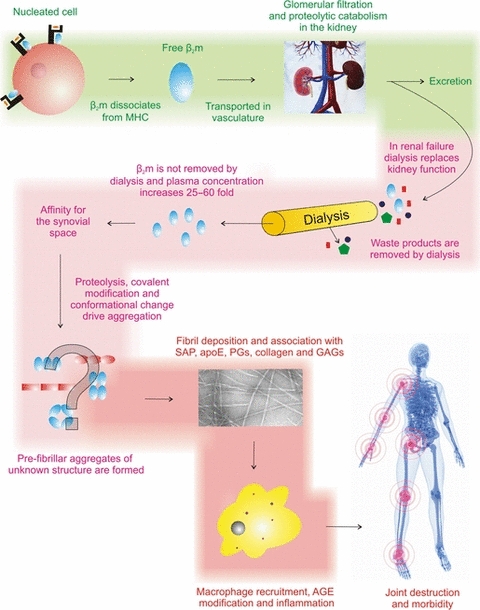
Schematic of the key processes which result in the pathological symptoms experienced in DRA (reproduced, with permission, from [138]).

Catabolism of β_2_m following its dissociation from the MHC I heavy chain occurs predominantly in the proximal tubules in the kidney [21,22]. As a consequence, the concentration of β_2_m circulating in the serum of patients suffering from renal dysfunction is enhanced up to 60-fold compared with healthy individuals. This causes the deposition of β_2_m as amyloid fibrils in osteoarticular tissues, leading to pathological bone destruction and the condition known as dialysis-related amyloidosis (DRA) (Fig. 2) [23]. However, a poor correlation between the β_2_m concentration in the serum and fibril load in osteoarticular tissues in long-term dialysis patients suggests that additional factors must be responsible for the initiation of β_2_m aggregation *in vivo* [24]. Consistent with these results, *in vitro* studies have shown that β_2_m is remarkably intransigent to assembly into amyloid fibrils at neutral pH, remaining predominantly monomeric for several months at pH 7.5, 37 °C, when incubated at protein concentrations more than 20-fold higher than those found in dialysis patients (∼ 3.2 μm [21]) [25,26]. As a consequence of these findings, factors have been sought that could facilitate protein aggregation of β_2_m *in vivo*, including the age of patients [27], the duration of kidney failure [28], the dialysis procedure itself [29–31], post-translational modifications of full-length β_2_m [32–40] and bimolecular collision between β_2_m and biological molecules abundant in osteoarticular tissues or encountered during dialysis [26,41–51]. As a result, a multitude of factors have been shown to enhance the aggregation of β_2_m *in vitro* and are implicated *in vivo*, including Cu^2+^ [47,52–59], glycosaminoglycans [26,41,60], lysophosphatidic acid [49], non-esterified fatty acids [48,50] and collagen [41,42,61].

Amyloid formation of β_2_m under physiological pH conditions (around pH 7.0) commences from the fully folded native protein state [62]. Analysis of the thermodynamic stability of native wild-type β_2_m and an array of variants, however, showed no correlation between the thermodynamic stability of β_2_m and its potential to assemble into amyloid-like fibrils *in vitro* [62]. Instead, the formation of one or more non-native precursors that are accessible by dynamic fluctuations from the native protein is required before aggregation can occur [9,18–20,63–69]. Such fluctuations may expose aggregation-prone sequences normally sequestered in the native structure [70], consistent with local and/or more global unfolding events being a common feature in the aggregation mechanisms of globular proteins [58,67,71–80].

## Peptidyl–prolyl isomerization initiates β_2_m amyloid assembly at physiological pH

In pioneering work, Chiti *et al.* [81] used a series of spectroscopic probes to show that wild-type β_2_m folds via two structurally distinct intermediates, known as I_1_ and I_2_, *en route* to the globular native state. The first intermediate along the folding reaction coordinate, I_1_, is populated within 5 ms of dilution of the protein from denaturant. This species shows substantial elements of non-random structure and contains a disorganized hydrophobic core in which several hydrophobic residues remain exposed to solvent [81]. The second folding intermediate, I_2_, forms within milliseconds of the population of I_1_ and displays native-like secondary structure and ordered packing of side chains within the hydrophobic core. Further folding of I_2_ occurs on a timescale of seconds to minutes at 30 °C, suggesting substantial energetic barriers to the attainment of the globular native fold [62,81]. Although folding of wild-type β_2_m is a cooperative process as judged by equilibrium denaturation [81], I_2_ nonetheless accumulates, reaching a population of about 14 ± 8% at equilibrium at pH 7.4, 30 °C, as judged by capillary electrophoresis [82]. Importantly, the concentration of I_2_ was found to correlate with the rate of elongation using seeds formed from *ex vivo* amyloid fibrils at pH 7.4, 30 °C, consistent with this native-like folding intermediate being directly (or indirectly via further conformational changes) capable of amyloid elongation [82]. A slow folding intermediate, reminiscent of I_2_, has also been described by others [34,83].

Building on the observations made by Chiti and colleagues [82], more detailed studies of the folding and unfolding mechanism of wild-type β_2_m, combined with mutagenesis of the sequence, demonstrated that the transition between the slow folding intermediate I_2_ and the native fold is rate limited by *trans* to *cis* isomerization of the His31-Pro32 peptide bond, which led to the kinetically trapped intermediate being termed I_T_ [67–69]. Consistent with these findings, folding studies of a variant of β_2_m in which Pro32 is replaced with Val using manual mixing experiments at low temperature (2.8–4.0 °C) monitored by CD and NMR revealed that the slow folding step is abolished, trapping β_2_m in a non-native species presumably with a *trans* His31-Val32 peptide bond [68]. Pro32 is highly conserved in β_2_m in different organisms [84] and *trans* to *cis* peptidyl–prolyl isomerization at this site has been shown previously to be responsible for the slow refolding commonly found in other immunoglobulin domains [85–91]. Interestingly, however, P32V β_2_m is not able to elongate amyloid fibrillar seeds *in vitro* or to nucleate fibril formation, suggesting that a *trans* His31-Xaa peptide bond is necessary, but not sufficient, to endow β_2_m with its amyloidogenic properties [68].

To gain a more detailed understanding of the kinetic folding mechanism of β_2_m and the role of different partially folded species in linking the folding and aggregation energy landscapes, Jahn and co-workers [67] analysed the folding and unfolding kinetics of β_2_m under an array of conditions, including analysis of the folding mechanism of the variant P32G. Using global analysis of the resulting kinetic data, the authors proposed a five-state model for the folding mechanism of wild-type β_2_m involving parallel folding pathways initiated from *cis* or *trans* His31-Pro32 in the unfolded state [67]. The five-state model has been challenged by Sakata and co-workers [69] who proposed that a simpler four-state model satisfies their obtained microscopic and macroscopic rates of β_2_m unfolding and refolding using chevron analysis. In particular, using their approach Sakata *et al.* were unable to detect spectroscopically the accumulation of the folding intermediate containing a native *cis*-His31-Pro32 peptide bond (I_C_), suggesting that this species is non-existent or populated to levels below the detection limit. Despite these differences, both folding models suggest that I_T_ is low but significantly populated under physiological conditions at equilibrium, consistent with the poor ability of wild-type β_2_m to elongate fibrillar seeds at neutral pH *in vitro* [26,67]. Replacement of Pro32 with glycine (P32G) resulted in a simple three-state folding mechanism in which an intermediate, presumably with a *trans* His31-Gly32 peptide bond akin to I_T_, accumulates during folding, reaching an equilibrium concentration of approximately 30% [67]. Importantly, by titrating the population of I_T_ populated at equilibrium for the wild-type protein and P32G by varying the solution conditions, Jahn *et al.* [67] showed that the population of I_T_ correlates with the rate of fibril elongation *in vitro*, suggesting that I_T_ is a key link between the folding and aggregation energy landscapes for this protein. This could occur directly by this species showing an ability to elongate amyloid seeds, or indirectly via further conformational excursions to other species accessible from this folding intermediate [9,20,66,67]. Interrogation of the conformational properties of P32G using NMR suggested large conformational changes involving residues in the BC- and FG-loops, the D-strand and the N-terminal region of the protein that presumably arise from the isomerization of Pro32 and subsequent partial unfolding of the protein [67]. These regions map precisely to the regions reported previously to be perturbed in the kinetic folding intermediate I_T_, suggesting a close structural relationship of the two species [67].

The intransigence of wild-type β_2_m to form amyloid fibrils when incubated for extended periods of time at neutral pH at concentrations substantially higher than those found *in vivo* [21,25,26] can be rationalized in light of the finding that the amyloidogenic precursor, I_T_, is both transiently sampled and maintained at low concentrations at equilibrium in the wild-type protein under ambient conditions [25,67,82]. In order to explore the thermodynamics and kinetics of amyloid assembly from β_2_m at physiological pH *in vitro*, therefore, a plethora of conditions have been used to increase the population of species akin (but not necessarily identical) to I_T_ at equilibrium. These include the addition of Cu^2+^ ions and urea [46,47,53,92], organic solvents [60,83], collagen [41,42], glycosaminoglycans or other biologically relevant factors [26,60,93], SDS or lysophospholipids [48–51,94]. Changes in the physicochemical environment, including ultrasonication [95], heat treatment [96], high salt and stirring/agitation [97], have also been employed. These apparently very different conditions have in common the principle that they perturb the equilibrium position of the *cis/trans* His31-Pro32 peptide bond and hence enhance the amyloidogenic potential of the wild-type protein [25]. Mutations in the N- and/or C-terminal regions of the sequence have also been shown to enhance amyloid formation of β_2_m at physiological pH [8,9,25,26,32,98,99], whilst other mutations that focus on the DE-loop region demonstrated variable effects on the thermodynamic stability of the protein depending on the amount of strain introduced [14,16,20,100,101]. DE-loop mutations such as D59P that introduce loop strain show a decreased folding free energy compared with the wild-type protein and an enhanced potential to aggregate, whereas a release of loop strain such as in W60G leads to super-stable variants which have reduced amyloidogenic features [13,14,16]. However, DE-loop cleavage variants such as ΔK58 or cK58 (which contain a specific cleavage at Lys58 with or without removal of Lys58, respectively) have been demonstrated to be highly aggregation-prone [34,102–104]. Together these studies are indicative of a fragile and delicate amino acid network required for the stabilization of the *cis* isomer at His31-Pro32 that is required both for binding to the MHC I heavy chain [16] and to maintain a soluble native structure for the monomeric protein.

## β_2_m assembly mechanisms at atomic resolution

**Fig. 3:**
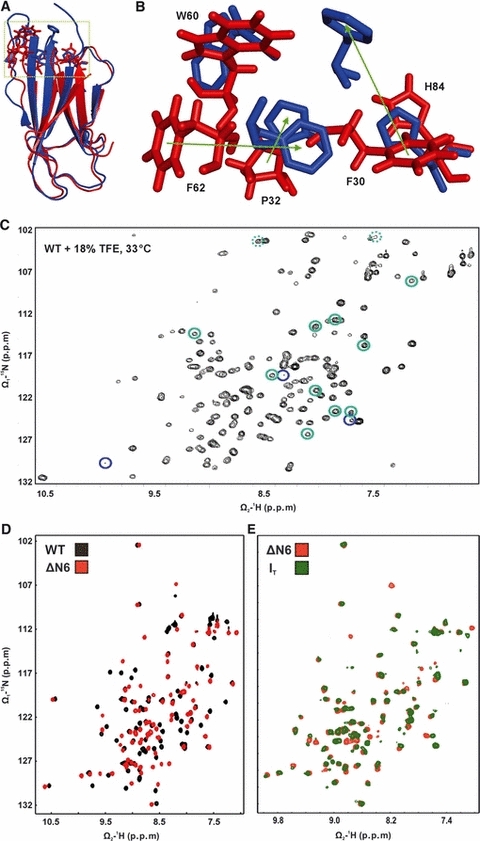
Molecular description of the I_T_ state using X-ray crystallography and high resolution solution NMR. (A) The ribbon overlay shows one monomer of the hexameric crystal structure of H13F (PDB code 3CIQ [55], in blue) and the lowest energy structure of ΔN6 (PDB code 2XKU) [9] (in red). The residues Phe30, Pro32, Trp60, Phe62 and His84 are highlighted in sticks. The dashed green box indicates a zoom-in for this region shown in (B). (C) ^1^H–^15^N HSQC of wild-type β_2_m in 18% (v/v) TFE at pH 6.6 and 33 °C (reproduced, with permission, from [20]). Green circles are assigned resonances for I_T_, while blue circles indicate the TFE induced, structurally disordered D state that is thought to be precursor for fibril elongation under these conditions. (D) ^1^H–^15^N HSQC overlay of wild-type β_2_m (black) and ΔN6 (red) recorded in 25 mm sodium phosphate buffer pH 7.5, 25 °C. (E) ^1^H–^15^N SOFAST HMQC overlay of ΔN6 (red) and the kinetic intermediate I_T_ (green) recorded approximately 2 min after refolding was initiated (25 mm sodium phosphate buffer pH 7.5, 0.8 m residual urea, 25 °C). Reproduced with permission from [9].

Clinical studies have shown that dialysis patients treated with Cu^2+^-free filter membranes have a > 50% reduced incidence of DRA compared with patients who were exposed to traditional Cu^2+^-containing dialysis membranes [27,105]. These studies suggest that Cu^2+^ ions may play a role in initiating or enhancing aggregation of wild-type β_2_m in DRA. Indeed, Cu^2+^ has been shown to bind to native human β_2_m with moderate affinity (*K*_app_ = 2.7 μm) and specificity (Cu^2+^ > Zn^2+^ >> Ni^2+^) [46,106]. Binding involves coordination to the imidizole ring of His31 [7,107]. Non-native states of wild-type β_2_m also bind Cu^2+^ ions; in this case the three other histidines in the sequence (His13, His51, His84) coordinate Cu^2+^ with a *K*_app_ ∼ 41 μm [107]. As a consequence, binding of Cu^2+^ ions increases the concentration of non-native (so-called ‘activated’) forms of monomeric β_2_m, named by Miranker and co-authors as M*, which triggers the formation of dimeric, tetrameric and hexameric species (< 1 h) believed to be on-pathway to amyloid-like fibrils [47,106]. Cu^2+^ binding is required for the conformational changes leading to the formation of M* and to the generation of early oligomeric species. However, once these oligomeric species and subsequent fibrillar aggregates are formed, Cu^2+^ is not essential for their stability [52,54,56,57,108]. By creating two variants, P32A and H13F, Miranker and colleagues [55,58] were able to crystallize dimeric and hexameric forms of β_2_m (the latter after Cu^2+^-induced oligomerization). These studies revealed that dimeric P32A and hexameric H13F contain a *trans* His31-Ala32 and a *trans* His31-Pro32 peptide bond, respectively. Each oligomer is composed of monomers that retain a native-like fold, yet display significant alterations in the organization of aromatic side chains within the hydrophobic core, most notably Phe30, Phe62 and Trp60 (Fig. 3A,B, in blue), which the authors speculate could be important determinants of amyloid assembly [53,55,58]. How these static structures relate to the transient intermediates formed during folding or populated during aggregation, however, remain unclear. Importantly in this regard, P32A and H13F lack an enhanced ability to assemble into amyloid fibrils compared with wild-type β_2_m [55,58], reminiscent of the behaviour of P32V [68,69]. Despite containing a *trans* His31-Xaa32 peptide bond, these species lack structural and/or dynamical properties critical for amyloid formation.

Increased conformational dynamics has emerged as a common feature of the assembly of β_2_m monomers into amyloid fibrils at neutral pH from a wealth of studies under varied solution conditions [9,10,18–20,32,65–67,92,103,109], akin to the findings on other proteins that also assemble into amyloid fibrils commencing from folded monomeric states [64,71,73,76,77,80,110–116]. Accordingly, ΔN6 (in which β_2_m is cleaved at Lys6) [32], cK58 and ΔK58 [34,102,103,117,118] and wild-type β_2_m in the presence of SDS/2,2,2-trifluoroethanol (TFE)/other additives [20,41,42,50,51,66,119] all exhibit decreased solubility, increased local and global unfolding events and enhanced amyloidogenicity at pH values close to physiological. Of particular interest is the variant ΔN6, since this species is found as a significant component (∼ 26%) in *ex vivo* amyloid deposits and exhibits an increased affinity for collagen compared with the wild-type protein, suggesting a role for this protein in the development of DRA [61,120]. Pioneering work by Esposito and colleagues showed that ΔN6 experiences a global decrease in conformational stability compared with wild-type β_2_m and, using molecular dynamics simulations, the authors proposed that the D-strand facilitates intermolecular interactions to form oligomeric assemblies prior to the development of long straight amyloid fibrils at pH 6.5, 37 °C [32]. Similarly, the variants cK58 and ΔK58 were found to be highly aggregation-prone, presumably due to enhanced conformational dynamics, especially for strand D, and a concomitant increase in concentration of the amyloidogenic folding intermediates at equilibrium [34,103]. In contrast, the mutation W60G which also lies in the DE-loop diminishes the potential of this variant to extend fibrillar seeds of the human wild-type protein at pH 7.4 in the presence of 20% (v/v) TFE [16], consistent with the dynamics within this region of the protein playing a crucial role in β_2_m assembly at neutral pH [13,14,19,20,66,121]. These studies therefore reinforce the importance of interrogating the conformational dynamics of β_2_m and its truncation variants in more detail in order to understand the aggregation properties of this species and, more generally, how other non-native species that retain a globular fold aggregate *in vitro* and *in vivo* [116].

Major breakthroughs in understanding the properties that endow non-native states of β_2_m with their amyloidogenic properties have arisen from NMR studies of wild-type β_2_m and several variants of the protein by exploiting the capabilities of modern NMR methods for rapid and sensitive data acquisition [7,9,11,20,32,55,58,66–68,103,109]. Accordingly, recent studies of the folding kinetics of wild-type β_2_m using real-time NMR combined with amino acid selective labelling of Phe, Val and Leu provided the first glimpses of the amyloid precursor of β_2_m under conditions close to physiological [109]. However, extensive peak broadening caused by conformational dynamics on a microsecond to millisecond timescale ruled out detailed assignment and structure elucidation of I_T_. Following on from this work, studies of the folding kinetics of wild-type β_2_m in different concentrations of TFE using real-time NMR revealed that the native protein is generated with double exponential kinetics from I_T_ for all resonances studied, indicative of an energy landscape that is more complex than the single barrier suspected hitherto [66,67,69]. By contrast with the behaviour of the wild-type protein, W60G folds to the native state from I_T_ with mono-exponential kinetics, indicative of a more simple folding energy landscape for this less amyloidogenic variant [66]. Based on these results, the authors propose that a species that is more disordered than I_T_ (named a ‘native-unlike’ or D state), formed maximally in 20% (v/v) TFE, is responsible for elongating wild-type β_2_m seeds [20]. The wild-type protein under those conditions has also been simulated using molecular dynamics [122]. Exploiting the sensitivity of β_2_m conformations to the concentration of TFE, the authors were able to find conditions wherein I_T_ is maximally populated from W60G, reaching 30–40% population in 18% (v/v) TFE (at pH 6.6, 33 °C), and were able to assign 63 backbone amide resonances (out of 93 amide bonds) unambiguously for this species (BMRB code 16587) (Fig. 3C) [20]. Incomplete assignment of the I_T_ state in W60G and considerable peak overlap by native state resonances, however, hampered the assignment of the backbone conformation of the peptidyl–prolyl bond at Pro32 and a more detailed structural and dynamic characterization of this intermediate [20].

**Fig. 4:**
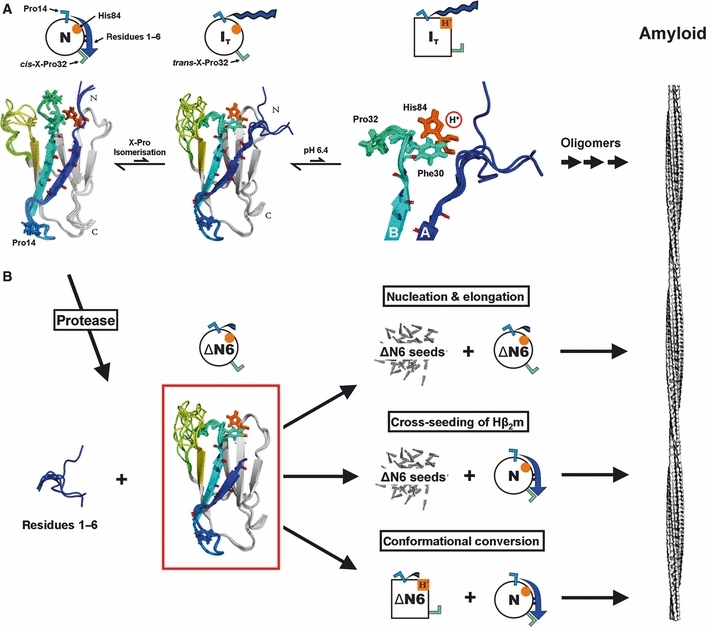
Prion-like conversion during amyloid formation. (A) Summary showing the structures of wild-type β_2_m (PDB code 2XKS) and a model of I_T_. Above, keys for these conformational states. Native wild-type β_2_m (leftmost), shown above as a circle with *cis* His31-Pro32 (green Γ), *trans* His13-Pro14 (blue Γ), His84 (orange circle) and the N-terminal region (residues 1–6, blue arrow). Backbone atoms of residues which establish strong hydrogen bonding between β-strands A and B in the native state are shown in sticks. Upon dissociation of the N-terminal region, the His31-Pro32 peptide bond is free to relax into the *trans*-conformation, causing further conformational changes that lead to the formation of the non-native I_T_ conformer (shown as a circle above a model of its structure). Protonation of His84 under mildly acidic conditions (shown in red ball and stick and as an orange square in the model above), which lies adjacent to Pro32, enhances the amyloid potential of I_T_ further. Oligomerization of these aggregation-prone species then leads to the formation of β_2_m amyloid fibrils. Assuming that the fibrils formed at neutral pH are structurally similar to those formed at acidic pH, as suggested by FTIR [135] and solid state NMR [133,134], large conformational changes are required in order to transform the anti-parallel β-sheet arrangement of ΔN6 into the parallel in-register arrangement of β-strands characteristic of β_2_m amyloid fibrils, as reported recently [132] (reproduced, with permission, from [9]). (B) Summary showing the consequences of β_2_m cleavage of the N-terminal hexapeptide that generates ΔN6 as a persistent I_T_ state (PDB code 2XKU). Once formed ΔN6 is able to nucleate and elongate its own fibrils and also to cross-seed elongation of its fibrillar seeds with the wild-type protein, leading to the development of long straight amyloid-like fibrils (the image of the fibrils was redrawn from the cryo-EM structure of β_2_m amyloid fibrils from [139]). Furthermore, ΔN6 can transform the innocuous native state of β_2_m via bimolecular collision. The formation of catalytic amounts of ΔN6 thus has been proposed to be a cataclysmic event during the development of DRA.

Most recently, the difficulties in determining the conformational properties of I_T_ have been overcome by using the β_2_m truncation variant ΔN6 as a structural mimic of this species (Fig. 3A,B, in red) [9,25]. High resolution NMR studies directly comparing the ^1^H–^15^N HSQC spectra of ΔN6 and I_T_ revealed that the major species populated by ΔN6 in solution at pH 7.5, 25 °C, closely resembles the transient folding intermediate I_T_ (Fig. 3D,E). Using ΔN6 as a structural model for I_T_, full resonance assignment and structural elucidation were possible, revealing the structural and dynamical properties of this non-native conformer of β_2_m. The results showed that under the conditions employed ΔN6 retains a native fold but undergoes a major re-packing of several side chains within the hydrophobic core to accommodate the non-native *trans*-conformation of the His31-Pro32 peptide bond (Fig. 3A,B, in red). Intriguingly, the side chains involved map predominantly to the same residues that undergo structural reorganization in the presence of Cu^2+^ ions, although the precise packing of residues remains different in many cases (Fig. 3A,B) [9,55,58]. Despite adopting a thermodynamically stable [9,25] native-like topology, ΔN6 is a highly dynamic entity, possessing only limited protection from hydrogen exchange together with pH- and concentration-dependent sensitivity of its backbone dynamics on a microsecond to millisecond timescale. These data suggest that increased conformational dynamics of ΔN6 correlate with an increase in its amyloidogenic properties presumably by enabling the formation of one or more rarely populated conformers that have an enhanced potential to assemble into amyloid fibrils [9,32,123]. One of the key events in this amyloid switch is protonation of His84, which experiences a large p*K*_a_ shift from ∼ 4 to ∼ 7 upon peptidyl–prolyl isomerization of the His31-Pro32 peptide bond (Fig. 4A) [9]. The involvement of His84 in the initiation of β_2_m amyloid fibril formation has been proposed previously using computational methods [61]. Oligomeric structures which become available after peptidyl–prolyl isomerization and exploration of conformational space upon His84 protonation have been proposed previously in association with Cu^2+^ binding [55,58], in the presence of dithiothreitol [124] or by the binding of nanobodies [125]. Interestingly, the last two conditions result in the formation of oligomers that are domain swapped, as proposed hitherto for β_2_m assembly under native conditions using computational methods [126] or Cu^2+^ treatment [106]. Whether domain swapping occurs in DRA, however, remains to be elucidated. Another open question is the structural and dynamic similarities and differences between *trans* intermediates formed under different conditions (such as alterations of pH and temperature, Cu^2+^ treatment, mutagenesis (ΔN6) or addition of organic solvent (TFE)) and how these map to the structure determined for ΔN6 at neutral pH [9] or that of the more ephemeral amyloid precursors that form from this protein or from the folding intermediate I_T_. Nonetheless, these data are suggestive of a mechanism of assembly under different solution conditions that contains many features in common.

## Prion-like conversion during β_2_m amyloid assembly

Despite the finding that ΔN6 comprises ∼ 26% of β_2_m in amyloid deposits in patients with DRA, this species is not found in the serum of people with renal dysfunction [127]. As a consequence of these findings, formation of ΔN6 has been proposed to occur as a post-assembly event [123]. Most recently, however, it has been demonstrated that ΔN6 is not only able to nucleate fibrillogenesis efficiently *in vitro* at physiological pH as discussed above (Fig. 4B) [9,25,26] but, as a persistent *trans*-Pro32 state, ΔN6 is also able to convert wild-type β_2_m into an aggregation-competent conformer by bimolecular collision between the two monomers (Fig. 4B) [9]. Accordingly, only catalytic amounts (1%) of ΔN6 are sufficient to convert significant quantities of the wild-type protein into amyloid fibrils (Fig. 4B). Detailed interrogation of bimolecular collision between native wild-type β_2_m and ΔN6 using NMR revealed the molecular mechanism by which this prion-like templating might occur [9]. First, ΔN6 binds specifically, but transiently, to native wild-type β_2_m, possibly involving residues of β-strands A, B and D and the DE-loop. This interaction changes the native configuration of Pro14 within the AB-loop which is highly dynamic as indicated by molecular dynamics simulations [63,122] and X-ray crystallography (Fig. 1C,D). Pro14 dynamics have been shown hitherto to be responsible for an alternative β_2_m conformation in which the hydrogen bonding between β-strands A and B is severely impaired [15]. Inter-strand hydrogen bonding between those two strands, together with the correct attachment of the N-terminal hexapeptide, has been demonstrated to be crucial in maintaining a low concentration of I_T_ at equilibrium [25]. Binding of ΔN6 to wild-type β_2_m, therefore, leads to the disruption of important interactions between the N-terminal hexapeptide and the BC-loop, leading to accelerated relaxation kinetics towards the amyloidogenic *trans* His31-Pro32 isomeric state. The truncation variant ΔN6 is thus capable of driving the innocuous native wild-type protein into aggregation-competent entities, reminiscent of the action of prions. Such an observation rationalizes the lack of circulating ΔN6 in the serum and, given the natural affinity of this species for collagen (which is enhanced relative to wild-type β_2_m [61]), explains why assembly of fibrils occurs most readily in collagen-rich joints. Rather than being an innocuous post-assembly event, therefore, proteolytic cleavage of β_2_m to create one or more species truncated at the N-terminus could be a key initiating event in DRA, enabling the formation of a species that is not only able to assemble *de novo* into amyloid fibrils but can enhance fibrillogenesis of wild-type β_2_m. The latter is accomplished by initiating the ability of the wild-type protein to nucleate its own assembly, or by cross-seeding fibril elongation of ΔN6 seeds with wild-type monomers (Fig. 4). Identifying the proteases responsible for the production of ΔN6 or using the high resolution structure of ΔN6 as a target for the design of small molecules able to intervene in assembly may provide new approaches for therapeutic intervention in DRA.

## Outlook: towards a complete molecular description of β_2_m amyloidosis

In this review we have highlighted the importance of conformational dynamics for the initiation and development of β_2_m amyloid formation commencing from the natively folded state. Detailed analysis of the folding, stability and amyloidogenicity of a number of different proteins has revealed that a polypeptide chain can adopt a diversity of structures within a multidimensional energy landscape, the thermodynamics and kinetics of which are dependent on the protein sequence and solution conditions employed [128]. One key feature that appears to identify amyloidogenic proteins from their non-amyloidogenic counterparts is a lack of structural cooperativity that is revealed by enhanced conformational dynamics on a microsecond to millisecond timescale, often portrayed by increased rates of proteolysis, hydrogen exchange and *R*_2_ NMR relaxation rates [115]. Such motions may expose sequences with high amyloid potential that are usually hidden within the native structure [70] or may endow surface properties that enable new protein–protein interactions to form. Studies of β_2_m have contributed substantially to this view, resulting most recently in a high resolution structure for the amyloid-initiating folding intermediate I_T_ and the beginnings of a molecular understanding of why increased conformational dynamics make this species highly aggregation-prone [9]. Rather than an innocuous post-assembly event, the work suggests proteolytic cleavage as a cataclysmic event that releases a species that is not only able to spawn further aggregation-prone species but is also able to convert the wild-type protein into an amyloidogenic state via conformational conversion akin to the activity famously associated with prions [129–131]. Finally, many studies of β_2_m amyloid assembly under a wide range of conditions, some close to physiological and others utilizing metal ions or solvent additives to drive fibrillogenesis at neutral pH, have together revealed common principles of β_2_m self-assembly which are related by the formation of non-native species initiated by a *cis* to *trans* His31-Pro32 switch despite the wide range of conditions employed. Further work is now needed to define the origins of molecular recognition between monomers and oligomers that form as assembly progresses into amyloid fibrils at neutral pH and to define the extent of further conformational changes required to form the cross-β structure of amyloid [132–135]. This will entail greater structural knowledge about the multitude of protein states populated on the folding and aggregation energy landscapes and how these species are formed and interconnected.

## Abbreviations

β_2_m: β_2_-microglobulin
DRA: dialysis-related amyloidosis
MHC I: major histocompatibility complex class I
TFE: 2,2,2-trifluoroethanol
